# Innovative Diagnostic Approaches and Challenges in the Management of HIV: Bridging Basic Science and Clinical Practice

**DOI:** 10.3390/life15020209

**Published:** 2025-01-30

**Authors:** Mohd Afzal, Shagun Agarwal, Rabab H. Elshaikh, Asaad M. A. Babker, Einas Awad Ibrahim Osman, Ranjay Kumar Choudhary, Suresh Jaiswal, Farhana Zahir, Pranav Kumar Prabhakar, Anass M. Abbas, Manar G. Shalabi, Ashok Kumar Sah

**Affiliations:** 1 Department of Medical Laboratory Technology, Arogyam Institute of Paramedical & Allied Sciences (Affiliated to H.N.B.Uttarakhand Medical Education University), Roorkee 247661, Uttarakhand, India; afzalglocal@gmail.com; 2 Shagun Agarwal, School of Allied Health Sciences, Galgotias University, Greater Noida 203201, Uttar Pradesh, India; shagunmpt@gmail.com; 3 Department of Medical Laboratory Sciences, College of Applied & Health Sciences, A’ Sharqiyah University, Ibra 400, Oman; rabab.mahmoud@asu.edu.om (R.H.E.); einas.osman@asu.edu.om (E.A.I.O.); 4 Department of Medical Laboratory Sciences, College of Health Sciences, Gulf Medical University, Ajman 4184, United Arab Emirates; azad.88@hotmail.com; 5 Department of Medical Laboratory Technology, Amity Medical School, Amity University Haryana, Gurugram 122412, HR, India; r.choudharymt@gmail.com; 6 School of Health & Allied Sciences, Pokhara University, Pokhara 33700, Nepal; suuress@gmail.com; 7 Department of Biology, College of Science, Qassim University, Buraidah 51452, Saudi Arabia; farhanazahir@gmail.com; 8 Parul Institute of Applied Sciences & Research and Development Cell, Parul University, Gujarat 391760, Vadodara, India; prabhakar.iitm@gmail.com; 9 Department of Clinical Laboratory Sciences, College of Applied Medical Sciences, Jouf University, Sakaka 72388, Saudi Arabia; anasseen@hotmail.com (A.M.A.); dr.mpathology@gmail.com (M.G.S.)

**Keywords:** biosensing techniques, HIV diagnosis, point-of-care systems, innovation, CRISPR

## Abstract

Human Immunodeficiency Virus (HIV) remains a major public health challenge globally. Recent innovations in diagnostic technology have opened new pathways for early detection, ongoing monitoring, and more individualized patient care, yet significant barriers persist in translating these advancements into clinical settings. This review highlights the cutting-edge diagnostic methods emerging from basic science research, including molecular assays, biosensors, and next-generation sequencing, and discusses the practical and logistical challenges involved in their implementation. By analyzing current trends in diagnostic techniques and management strategies, we identify critical gaps and propose integrative approaches to bridge the divide between laboratory innovation and effective clinical application. This work emphasizes the need for comprehensive education, supportive infrastructure, and multi-disciplinary collaborations to enhance the utility of these diagnostic innovations in improving outcomes in patients with HIV.

## 1. Introduction

Human Immunodeficiency Virus (HIV) continues to represent a significant global health burden, impacting millions of people and causing life-threatening immune system breakdown if not treated. Despite advances in antiretroviral treatments (ARTs), which have increased the survival rates and quality of life of patients with HIV, quick diagnosis and persistent monitoring are still required for optimal disease management. Early and precise diagnosis enables rapid action, lowering the viral load, halting progression to Acquired Immune Deficiency Syndrome (AIDS), and restricting transmission [1].

Traditional HIV testing methods, though reliable, sometimes fall short in resource-limited situations, and their sensitivity and specificity vary depending on the stage of infection. Diagnostic technology advancements, such as molecular assays, biosensors, and next-generation sequencing, provide new opportunities for improving accuracy, speed, and accessibility. However, translating these advances into clinical practice offers substantial hurdles, emphasizing the need for greater convergence between fundamental science and healthcare delivery [2].

The purpose of this study is to examine current developments in HIV diagnostics, evaluate the clinical difficulties posed by these technologies, and suggest methods for successfully incorporating them into clinical settings. This review aims to address real-world challenges in infrastructure, training, and resource allocation while highlighting the promise for improving patient outcomes through an examination of molecular, biosensor, sequencing, and point-of-care diagnostic technologies. In order to direct future research and make it easier to integrate diagnostic advancements into routine HIV management procedures, the scope of this study also includes defining the multidisciplinary strategies and regulations required to overcome these obstacles while emphasizing the importance of cost-effective diagnostic innovations to enhance accessibility in resource-limited settings and improve global HIV management.

## 2. Methodology

### 2.1. Search Strategy

A comprehensive literature search was conducted across major scientific databases, including PubMed, Scopus, Web of Science, and Google Scholar, to gather relevant studies published in the past decade on advancements in HIV diagnostic approaches. The search terms included combinations of keywords such as “HIV diagnostics”, “molecular assays”, “biosensors”, “next-generation sequencing”, “point-of-care testing”, “clinical translation”, and “implementation challenges”. Filters were applied to include only peer-reviewed articles, review papers, and clinical guidelines published in English. Studies were further selected based on relevance, focusing on innovations in HIV diagnostics, barriers to clinical implementation, and strategies for bridging the gap between laboratory research and clinical application. Additional sources were reviewed from the references of selected papers to ensure comprehensive coverage of emerging technologies and practical challenges in HIV management.

### 2.2. Study Selection

**Figure 1:**
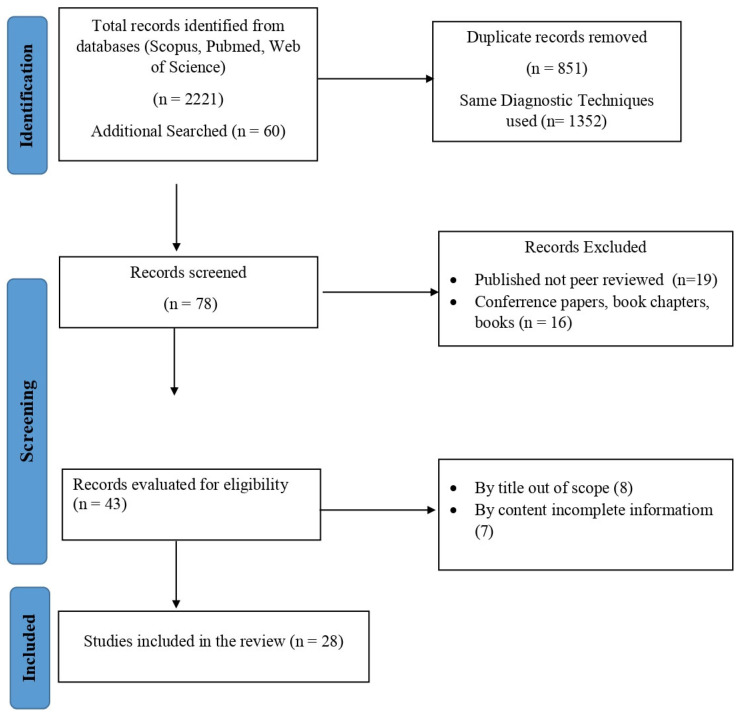
Illustrates the structured flowchart used to outline the study selection process for this review.

The selection criteria for this review required studies to be published between 2012 and 2024. Eligible articles included only meta-analyses, clinical trials, and systematic reviews, ensuring the inclusion of high-quality evidence. Additionally, all studies had to be published in English and undergo peer review to maintain research reliability. The articles selected focused on advancements in HIV diagnostics, barriers to clinical implementation, and strategies for bridging the gap between laboratory research and clinical application. Studies that did not meet these requirements, such as conference abstracts, opinion pieces, or non-peer-reviewed publications, were excluded from consideration (Figure 1).

## 3. Diagnostic Innovations in HIV

Table 1 presents advancements in HIV diagnostic techniques, outlining the progression from foundational to advanced methods with corresponding references and estimated time periods.

**Table 1:** This table highlights advancements in diagnostic techniques for HIV, progressing from foundational to advanced methods, along with references and approximate time periods for each.

Diagnostic Technique	Description	References	Time Period
ELISA (Enzyme-Linked Immunosorbent Assay)	Detects HIV antibodies in blood samples, providing first lab-based serological test for HIV	[3]	Early 1980s
Western Blot	Confirmatory test for HIV, identifying specific HIV proteins via antibody binding	[4]	Mid-1980s
Rapid Antibody Tests	Quick detection of HIV antibodies using fingerstick blood or oral fluids, e.g., OraQuick HIV test	[5]	Early 2000s
NAT (Nucleic Acid Testing)	Directly detects HIV RNA in blood, useful for early detection and confirmation	[5]	2000s
PCR (Polymerase Chain Reaction)	Identifies HIV DNA/RNA in blood, especially valuable in early detection and viral load assessment	[6]	1990s
qPCR (Quantitative PCR)	Quantifies HIV viral load in blood to monitor treatment effectiveness and disease progression	[7]	Early 2000s
Multiplex Testing	Combines HIV antibody and antigen detection to increase sensitivity, identifying both acute and chronic infections	[8]	2010s
Lab-on-a-Chip and Microfluidics	Miniaturized diagnostics integrating multiple assays for rapid POC HIV testing, e.g., CD4+ counts	[9]	2010s–present
NGS (Next-Generation Sequencing)	High-throughput sequencing allowing detailed HIV genetic analysis, detecting drug resistance and viral diversity	[10]	Late 2000s–present
Biosensors	Detects HIV antigens/antibodies or nucleic acids with portable sensors for POC, enabling rapid results	[11]	2010s–present
CRISPR-Based Diagnostics	Gene-editing technology adapted to detect HIV nucleic acids with high sensitivity, e.g., SHERLOCK assay	[12]	2016–present
Machine Learning and AI	Analyzes large genomic datasets to predict HIV drug resistance patterns, optimizing treatment regimens	[13]	2020s–present

### 3.1. Molecular Assays

Molecular assays have become essential in HIV diagnoses due to their excellent sensitivity and specificity, particularly in identifying low viral loads and enabling early diagnosis. Advances in molecular diagnostics, such as polymerase chain reaction (PCR) and real-time quantitative PCR, have greatly improved the ability to detect and quantify HIV RNA in clinical samples. These technologies enable accurate diagnosis, even in early-stage or acute HIV infections, where antibody-based testing may be ineffective [14].

#### 3.1.1. Polymerase Chain Reaction (PCR) Developments

Polymerase chain reaction (PCR) has advanced significantly in HIV diagnosis, mostly through improvements that improve sensitivity and adaptability to clinical circumstances. PCR enables the amplification of HIV-specific nucleic acids, which allows the virus to be detected before the immune system produces antibodies. This is especially important in cases of acute HIV infection, because early treatment can prevent rapid disease development and lower transmission rates. PCR-based diagnostics can now detect and quantify HIV RNA with high specificity, providing a strong tool for monitoring viral load in patients undergoing antiretroviral therapy (ART). Advances in PCR technology, including nested PCR and ultra-sensitive PCR tests, have enhanced the detection of viral reservoirs, thereby adding to research on HIV latency and prospective curative techniques [15].

#### 3.1.2. Real-Time Quantitative PCR (qPCR)

Real-time quantitative PCR (qPCR) has enhanced the use of molecular tests in HIV management. Unlike conventional PCR, qPCR enables the real-time monitoring of amplified DNA, yielding both qualitative and quantitative information about viral load in a single test. This quantitative feature is crucial in guiding ART since it measures treatment efficacy and monitors medication resistance. qPCR is also highly sensitive, capable of identifying trace amounts of viral RNA, increasing HIV detection accuracy, and reducing false negative results. qPCR technology has evolved to accommodate high-throughput testing, which is critical for large-scale epidemiological research and addressing high-demand environments such as urban clinics and hospitals in HIV-endemic areas [16]. Quantitative PCR (qPCR) is important in HIV-1 cure research because it enables accurate detection and quantification of the virus’s latent reservoir, which persists even after ART. Q4PCR, a unique multiplex qPCR technique, targets four separate sections of the HIV-1 genome in a single reaction: the packaging signal (PS), gag, pol, and env. This approach improves sensitivity and specificity by validating positive results using next-generation sequencing (NGS). Combining the qPCR and NGS techniques provides a high-throughput, unbiased reservoir analysis, which is critical for evaluating and developing curative options for HIV-1 elimination or silence [7]. Advances in molecular qPCR constitute a huge step forward in HIV diagnosis. To fully realize the potential of these technologies, problems such as cost, infrastructure needs, and the need for skilled staff must be addressed, particularly in resource-constrained environments. Overcoming these limitations will be critical to increasing the availability of these tests and guaranteeing successful HIV management worldwide.

#### 3.1.3. Nucleic Acid Testing (NAT)

Nucleic Acid Testing (NAT) plays a pivotal role in the diagnosis of HIV by detecting the virus’s genetic material (RNA or DNA), allowing for earlier detections than conventional antibody-based methods. NAT can identify HIV infection within days, even during the “window period” before antibodies are detectable. This early detection capability is critical for timely intervention, especially since individuals with acute HIV infection exhibit high viral loads and are more likely to transmit the virus. NAT also offers high sensitivity and specificity, ensuring accurate results and minimizing false positives and negatives, which is particularly important in blood screening to prevent transfusion-transmitted infections [17].

Beyond initial diagnosis, NAT is essential for monitoring viral load in patients with HIV undergoing antiretroviral therapy (ART), providing data on treatment efficacy by measuring viral RNA levels in the bloodstream. Additionally, NAT can detect various HIV subtypes, enhancing diagnostic accuracy in regions with diverse strains. This adaptability is also vital in high-risk populations, such as individuals on pre-exposure prophylaxis (PrEP) or those recently exposed, where NAT’s sensitivity can detect low-level infections often missed by standard tests. For infants born to mothers who are HIV positive, NAT is indispensable as it bypasses interference from maternal antibodies, enabling early diagnosis and prompt treatment [7].

### 3.2. Biosensors and Lab-on-a-Chip Technologies

Biosensors and lab-on-a-chip technologies have made significant advances in HIV diagnoses by allowing rapid, sensitive, and portable testing. These systems integrate biological detecting components with electrical or optical sensors, enabling the identification of HIV biomarkers such as antigens and antibodies in small sample volumes. Lab-on-a-chip technology reduces complex laboratory processes to a single chip capable of carrying out several experiments at the same time. This strategy is especially effective in low-resource environments where typical lab facilities are restricted, as it shortens the testing time, lowers costs, and eliminates the need for specialized equipment. Recent advances have resulted in biosensors that can detect low viral loads with excellent specificity, making them ideal for early HIV diagnosis and surveillance in a variety of clinical and field situations [18].

Researchers have leveraged microfluidic technology within lab-on-a-chip platforms to enhance point-of-care (POC) HIV diagnostics with promising outcomes. Mauk et al. demonstrated successful applications of microfluidics for HIV POC testing [19]. Glynn et al. introduced a microfluidic chip that uses CD4+ cell counts, utilizing magnetophoresis to detect HIV infection and AIDS [20]. This chip, with a high capture efficiency of 93%, enables manual operation without requiring an external pump, thus simplifying its use in diverse settings. Similarly, Liu et al. developed a microfluidic chip based on CD4+ cell counts combined with immunomagnetic separation. Unlike traditional methods, this chip quantifies CD4+ cells by DNA content, yielding precise results from as little as 10 μL of whole blood, closely matching standard flow cytometry analysis. Following this approach, the Alere Pima™ CD4 system was introduced in 2010, delivering reliable CD4+ counts within 20 min, demonstrating the lab-on-a-chip technology’s potential to streamline HIV diagnostics and expand access to essential testing in various healthcare environments [21].

### 3.3. Next-Generation Sequencing

Next-generation sequencing (NGS) has changed HIV research and diagnoses by allowing a thorough investigation of the viral genome. Unlike traditional sequencing approaches, NGS offers high-throughput, accurate, and cost-effective sequencing, allowing researchers to analyze vast parts of the HIV genome at once. NGS is effective at detecting small viral changes and drug-resistant viruses, making it useful for personalized treatment planning and efficacy monitoring [22].

Emerging diagnostic technologies, including nanotechnology, microfluidics, omics sciences, NGS, genomics big data, and machine learning, hold significant promise for achieving the UNAIDS 95-95-95 targets to end the HIV epidemic by 2030. These innovations encompass multiplexed diagnostics like biomarker-based point-of-care tests, molecular platforms, combination antibody–antigen assays, dried blood spot testing, and self-testing methods. Although antibody-based rapid tests have dominated HIV diagnostics since the first test was developed in the mid-1980s, newer targets such as nucleic acids and genes are now leveraged in nanomedicine, biosensors, microfluidics, and omics approaches, enabling earlier and more precise HIV detection. These technologies are favored for their ease of use, high diagnostic accuracy, speed, and ability to detect HIV-specific markers. However, further clinical and implementation research is needed to validate these approaches, and a public health framework will be essential to overcome clinical and operational challenges for their broader deployment [14].

### 3.4. Point-of-Care Testing: Rapid Antigen and Antibody Testing

Point-of-care (POC) testing has grown in popularity in HIV diagnostics due to its ease of use and quick turnaround time. Rapid antigen and antibody tests are commonly employed at the point of service to screen and diagnose HIV infection. These tests detect the HIV p24 antigen and/or antibodies and often give results within 15–20 min. The dual-detection capability boosts sensitivity, allowing for earlier diagnosis than antibody-only assays. POC tests are especially useful in community and rural health settings, where quick results enable timely counseling, referral to care, and ART initiation [23].

The use of oral fluids to detect HIV antibodies is a handy and non-invasive sampling procedure that improves patient acceptability. The US FDA has approved many lateral flow immunoassays (LFIAs), including the OraQuick Advance Rapid HIV-1/2 antibody test, for detecting HIV antibodies in a variety of sample types, including fingerstick blood, venipuncture blood, plasma, and oral fluids. OraQuick, which was first approved for professional use in 2004, was later certified for over-the-counter use with oral fluid in 2012, giving it an enticing choice for those who prefer private testing at home rather than public health facilities. Additionally, oral fluid sampling reduces healthcare workers’ exposure to bloodborne infections [24].

Although fast oral fluid-based HIV tests are useful, they have limitations. According to a meta-analysis, utilizing oral fluid reduced the sensitivity of the OraQuick test by about 2% compared to using fingerstick blood [25]. According to a Nigerian cohort study employing the Avioq HIV-1 Microelisa technology, there is also less sensitivity in detecting antibodies in oral fluid, particularly shortly after HIV infection. The study discovered that among 14 seroconverters, 35.7% exhibited consistent findings between plasma and oral fluid at all time points, whereas 64.3% had plasma reactivity before oral fluid specimens during early infection. The median delay between plasma and oral fluid reactivity was 29 days (*p* < 0.0039), with a significant difference of 69.5 days compared to RNA testing. Delayed antibody responses in oral fluid testing were detected independent of viral load or HIV subtypes, indicating that oral fluid testing is less sensitive than plasma-based techniques in the early stages of HIV infection. These findings highlight the limits of oral fluid testing in identifying HIV during early infection, particularly in individuals at increased risk of incidental HIV infection, and they highlight the need for caution when employing oral fluid testing in such settings [26]. The OraQuick test may miss HIV-1 infections on occasion due to poorer sensitivity than blood-based laboratory tests and variances in operator proficiency. Rapid HIV antibody testing may also fail to detect acute, early infections when the transmission risk is the greatest [24]. Despite these limitations, the OraQuick oral fluid test underscores the LFIA platform’s potential for non-blood sample testing in home and resource-limited settings, similar to home pregnancy or drug abuse tests using urine.

### 3.5. CRISPR-Based Diagnostics for HIV

CRISPR-based HIV diagnostics have emerged as a viable tool because of their high sensitivity, specificity, and flexibility. The CRISPR (Clustered Regularly Interspaced Short Palindromic Repeats) system, which is usually used for gene editing, can be modified to recognize specific nucleic acid sequences. CRISPR technology has been used in HIV diagnostics with systems such as CRISPR-Cas12 and CRISPR-Cas13, which can target and bind to HIV RNA or DNA sequences, triggering collateral cleavage activity that provides a detectable fluorescence signal when the viral genome is present [12].

The SHERLOCK (Specific High-Sensitivity Enzymatic Reporter UnLOCKing) platform, which is based on CRISPR-Cas13a, is an outstanding example. SHERLOCK detects small amounts of viral RNA by first amplifying the target nucleic acid with recombinase polymerase amplification (RPA) and then cleaving it with Cas13. This approach has demonstrated great sensitivity and specificity in identifying HIV, even at low viral loads, making it appropriate for early-stage infection diagnosis [27].

Another technique, the DETECTR (DNA Endonuclease-Targeted CRISPR Trans Reporter) technology, uses Cas12 to selectively target HIV DNA sequences. This technology has shown comparable sensitivity and is especially suitable for point-of-care (POC) testing, giving immediate, real-time findings. The Cas12-based approach was examined for detecting HIV in a range of clinical samples, and its ability to rapidly distinguish between distinct viral strains was a significant advantage for resource-limited situations [28].

Recent research has also focused on merging CRISPR diagnostics with microfluidics to produce lab-on-a-chip devices capable of automated sample processing and readout. This integration is intended to make CRISPR diagnostics more accessible and user-friendly, particularly in low-resource situations where standard laboratory facilities may be constrained [29].

Overall, CRISPR-based HIV diagnostic approaches have the potential to transform HIV testing by making it faster, cheaper, and more accessible while retaining high accuracy. However, clinical validation and regulatory approval are still needed before widespread adoption [30].

### 3.6. Machine Learning and AI for HIV Diagnosis

Machine learning (ML) and artificial intelligence (AI) are rapidly being used in HIV diagnostics to improve detection accuracy, forecast disease progression, and optimize treatment regimens. These tools can find patterns and anticipate outcomes that standard diagnostic procedures may not detect.

#### 3.6.1. Predictive Modeling for HIV Diagnosis and Progression

Machine learning models have been used to accurately predict HIV diagnosis and disease progression by analyzing electronic health records (EHRs), genetic data, and patient history. Wiewel et al. used machine learning algorithms to analyze data on patients with HIV and forecast which patients will have quicker disease progression based on baseline features and biomarkers. These models can aid doctors in making personalized treatment decisions and implementing early intervention measures for patients which are at risk [31].

#### 3.6.2. Diagnostic Accuracy and Rapid Screening

AI and machine learning (ML) approaches are improving the speed and accuracy of HIV tests, particularly in point-of-care settings. Image recognition models may be used to automate the examination of diagnostic test findings, such as lateral flow assays (LFAs), by accurately analyzing test lines and deciding whether the results are positive or negative. Studies have demonstrated that these machine learning-driven solutions can even beat human interpretation in terms of precision, helping to eliminate human error and enable precise diagnosis in resource-limited environments [32].

#### 3.6.3. AI in HIV Screening and Early Detection

AI has also shown potential in diagnosing early HIV infection by analyzing patterns in standard blood test data, which are typically accessible before particular HIV tests are performed. This technique has the potential to increase early diagnosis rates, particularly in high-risk populations, by identifying instances that require further testing. Barrios et al. used machine learning algorithms for normal lab data to discriminate between early HIV infection and other non-HIV illnesses, minimizing diagnostic delays and perhaps restricting transmission [14].

### 3.7. Limitations in HIV Diagnostic Approaches

HIV diagnostic techniques have drawbacks, such as sensitivity gaps, where early infections or low viral loads go unnoticed, and latency difficulties, which delay diagnosis owing to the detection window. Furthermore, resource restrictions such as high expenditures, infrastructure demands, and requirements for qualified people impede accessibility, particularly in resource-limited areas [33].

#### 3.7.1. Sensitivity Gaps

Many diagnostic methods, such as ELISA and Rapid Antibody Tests, are unable to identify HIV during the acute phase due to low antibody levels in the detection window period, resulting in false negatives. Traditional approaches, such as Western blot, also struggle to detect HIV in early infections, resulting in delays in proper diagnosis. Even modern techniques, such as NAT and qPCR, may have difficulty detecting the virus when the viral load is exceedingly low, particularly in individuals on antiretroviral treatment (ART) [34].

#### 3.7.2. Latency Issues

Most antibody-based tests, such as ELISAs and rapid tests, rely on the development of detectable antibodies, which might take weeks after infection, causing delays in diagnosis. Advanced technologies such as PCR and NGS need extensive processing periods, which further delay diagnosis and treatment. Additionally, existing diagnostic methods have difficulty finding latent HIV in viral reservoirs, which is important for effective treatment and cure programs [33].

#### 3.7.3. Resource Constraints

Advanced diagnostics, such as qPCR, NGS, and CRISPR-based technologies, are prohibitively expensive, limiting access in low-income areas, whereas tools such as NAT and multiplex testing necessitate sophisticated laboratory infrastructure, dependable electricity, and trained personnel, which are frequently unavailable in underserved communities. Dependence on specialized chemicals complicates logistics and raises costs, while technologies such as machine learning-based diagnostics and biosensors need highly qualified workers for operation and maintenance [35].

#### 3.7.4. Cost-Effective Diagnostic Innovations for Resource-Limited Settings

Cost-effective and scalable diagnostics, such as point-of-care (POC) testing, provide speedy and reliable findings without the need for specialized laboratories [36]. Affordable technologies such as microfluidic assays and isothermal amplification have demonstrated promise for low-cost HIV detection, particularly when linked with public health programs to increase early detection and monitoring. Additionally, diagnostics capable of detecting diverse HIV subtypes ensure equitable access to accurate results worldwide. These advancements are vital for achieving global HIV elimination goals [1].

## 4. Challenges in Clinical Translation

The clinical translation of improved HIV diagnostics confronts a number of difficulties that limit their inclusion into ordinary practice. These include logistic, economic, and infrastructure challenges, as well as concerns about patient accessibility, acceptability, and training providers. Each of these barriers has a significant impact on the practical use of innovative technology in real-world contexts [37].

### 4.1. Logistic Barriers

Logistic challenges in deploying sophisticated HIV diagnostic methods include the need for specialized equipment, storage needs, and supply chain consistency, particularly in resource-constrained situations. Certain diagnostic methods, such as point-of-care (POC) nucleic acid amplification assays, are challenging to scale in places without reliable electricity or refrigeration. Furthermore, problems with sample transit and processing in remote places cause delays and probable deterioration, compromising test accuracy and rapid diagnosis [36]. To overcome these constraints, studies emphasize the importance of regionally specialized logistic planning and resource allocation [5].

Managing Logistic Barriers in HIV Diagnostics

Table 2 illustrates a holistic strategy for addressing the logistic challenges commonly encountered in HIV diagnostic services.

Managing logistic hurdles in HIV testing is crucial to improving patient outcomes and meeting public health objectives. Common logistic problems include estimating diagnostic requirements, obtaining testing kits and equipment, and ensuring a steady supply chain. Strategies for overcoming these hurdles are being introduced in high-burden regions, frequently with the help of initiatives such as PEPFAR (President’s Emergency Plan for AIDS Relief). These activities are aimed at anticipating supply demands, optimizing procurement procedures, and guaranteeing the safe storage and efficient delivery of HIV tests. Effective supply chain management systems, such as those created by Global Health Supply Chain (GHSC) programs, are required to meet diagnostic demand. These systems use technology that enables the real-time tracking of HIV diagnostic materials at many levels, ranging from central warehouses to health clinics. Furthermore, data-driven decision-making technologies help to prevent stockouts by allocating resources based on demand and predicted usage rates [5,36].

### 4.2. Economic and Infrastructural Constraints

Economic constraints limit access to high-cost diagnostic tools, since healthcare funds in many places prioritize other pressing health needs. Furthermore, maintaining diagnostic infrastructure, which includes equipment and reagents for procedures such as next-generation sequencing and CRISPR-based diagnostics, can be prohibitively expensive. According to Zhang et al., modern diagnostic instruments frequently demand large initial investments, and the expense of operation and maintenance might strain limited resources, rendering them unsuitable for long-term usage in low- and middle-income nations [38]. Infrastructure deficiencies in laboratory facilities, particularly in rural locations, restrict the scalability of these technologies [39].

### 4.3. Patient Accessibility and Acceptability Issues

Ensuring that patients have access to and are willing to employ new diagnostics is a major hurdle. Diagnostics that need several visits to the clinic or sophisticated procedures are less likely to be accepted by patients, especially in rural locations where travel to healthcare facilities can be costly and time-consuming. Additionally, the stigma associated with HIV testing may discourage people from seeking care [40]. For example, Zeleke et al. found that patients preferred home-based testing and self-testing owing to privacy concerns, but the lack of understanding about these alternatives remains a barrier [41].

### 4.4. Education and Training Gaps

Healthcare professionals must be educated to utilize and interpret data from innovative diagnostic instruments for successful clinical deployment, which can be difficult in resource-constrained areas. Training gaps are widespread in developing technologies such as molecular diagnostics and point-of-care testing, where a shortage of competent workers can result in improper use and interpretation of data. According to Ávila-Ríos et al., continual education and organized training programs are required to ensure that healthcare practitioners are proficient in the operational and interpretative components of sophisticated HIV diagnoses. Without properly educated workers, the benefits of these technologies cannot be fully realized, and healthcare results may suffer [22].

Addressing these challenges needs a multidimensional approach that includes investments in infrastructure, training, and community engagement, as well as legislative support, to make modern HIV tests more accessible and sustainable in a variety of healthcare settings [42].

## 5. Bridging the Gap: Strategies for Integration

Innovative HIV diagnostics must be integrated into healthcare systems through strategic efforts that include cross-disciplinary collaboration, infrastructure and technology investment, comprehensive healthcare training, and supporting public health policies. Addressing each of these components can help to fill the gap between technological developments and practical, accessible HIV care [43] (Table 3).

**Table 3:** This table provides an overview of key strategies for bridging the gap in HIV diagnostics, focusing on integrating innovative approaches into healthcare systems.

Strategy	Description	References
Interdisciplinary Collaboration	Collaboration among healthcare providers, researchers, and policymakers fosters innovation and translates research into clinical practice.	[44]
Infrastructure and Technological Investment	Building diagnostic infrastructure and investing in technologies like point-of-care testing and mobile health units expand reach and reliability.	[45]
Healthcare Professional Training and Education	Equipping healthcare workers with updated skills through training in diagnostics, patient handling, and emerging technologies enhances service delivery.	[46]
Public Health Policies and Supportive Frameworks	Establishing supportive policies, including funding for diagnostics and patient access programs, ensures sustainable health outcomes and continuity of care.	[47]

### 5.1. Interdisciplinary Collaboration

Interdisciplinary collaboration among scientists, doctors, public health authorities, and politicians is required to integrate novel HIV tests. Collaboration enables the translation of research results into healthcare solutions that address real-world requirements. According to Grossman et al., strong collaborations between laboratory scientists and clinicians have accelerated the adoption of diagnostic innovations, such as molecular-based HIV tests, by enabling real-time feedback and ensuring that technologies are appropriate for a variety of healthcare settings [44]. This collaborative approach allows for the customization of diagnostics to meet the demands of certain populations, such as those in resource-limited areas.

### 5.2. Infrastructure and Technological Investment

To adopt HIV diagnostics effectively, healthcare institutions must have infrastructure that supports modern technologies like next-generation sequencing and AI-driven tools. Investments in strong laboratory infrastructure, data storage, and internet connectivity are critical, especially in low- and middle-income nations. Han et al. emphasized the need for long-term technical investment in diagnostic facilities, which improves access to and accuracy of HIV testing over time [45]. Furthermore, donor funding and government expenditures can help underserved regions deploy and sustain innovative diagnostic technology.

### 5.3. Healthcare Professional Training and Education

A skilled staff is essential for efficiently implementing novel HIV diagnoses. Training programs must be established to give healthcare personnel the skills necessary for sophisticated diagnostics, such as data interpretation for AI-assisted testing. Kennedy et al. discovered that the quality of training offered to healthcare workers has a substantial impact on the effectiveness of novel HIV detection methods, which require continual education to keep up with fast advancing technology [46]. Training programs customized to local healthcare contexts can help encourage long-term integration by providing professionals with the appropriate skills and knowledge.

### 5.4. Public Health Policies and Supportive Frameworks

Supportive public health policies are critical in the integration of innovative diagnostics, encouraging wider usage through regulatory backing and financing. Policies that support new diagnostics in regular HIV screening and monitoring can help assure that these techniques are included in standard care practices. UNAIDS and the World Health Organization have both said that supporting frameworks should include incentives for diagnostic innovation, expediting regulatory processes, and encouraging global health collaborations [47]. Public health policies can also help to remove obstacles to access by promoting equal allocation of resources and diagnostics in underprivileged populations.

## 6. Future Directions and Emerging Technologies

Future trends in HIV diagnosis and treatment focus on creating technologies that are quicker, more accurate, and usable in a wide range of healthcare settings. Emerging technologies are poised to overcome present obstacles in early identification, monitoring, and treatment response, all while enabling decentralized and patient-centered care. CRISPR-based diagnostics, AI-powered diagnostic algorithms, and biosensor technology developments are among the key innovations in HIV diagnoses and therapy that are being investigated [48,49].

### 6.1. CRISPR-Based Diagnostics

CRISPR technology, which was originally designed as a tool for gene editing, has now evolved into diagnostics with the promise for high specificity and sensitivity in HIV detection. CRISPR-based diagnostic tools may identify particular sequences of HIV RNA or DNA in patient samples, allowing for fast identification of the virus even at low levels. These techniques are useful for tracking HIV reservoirs and detecting growing treatment resistance, making them a viable tool for personalized care. As CRISPR diagnostic techniques improve, their low cost and quick outcomes may make them useful in point-of-care (POC) settings in resource-constrained locations [50].

### 6.2. AI-Powered Diagnostic Algorithms

Artificial intelligence (AI) has shown great promise for analyzing massive datasets and detecting patterns that might improve HIV diagnosis. Machine learning algorithms based on clinical and genomic data may enhance early detection and treatment outcomes, allowing for more tailored therapies. AI applications in HIV research include discovering genetic indicators for disease progression, aiding in medication development, and improving clinical decision-making procedures. Research is undertaken to guarantee that AI models can be installed on portable devices and are compatible with proof-of-concept testing, making diagnostics more accessible [51].

### 6.3. Advancements in Biosensor Technology

Biosensors, particularly lab-on-a-chip (LOC) technology, continue to progress, allowing for quick and portable HIV testing. These miniature platforms enable the very sensitive detection of HIV antibodies, antigens, and viral RNA/DNA from tiny samples, such as blood and oral secretions. Emerging LOC technologies are intended to be user-friendly and operable by non-specialists, with accurate findings delivered in less than an hour. With further improvement, biosensors can enable at-home HIV testing and enhance diagnosis in rural areas where access to healthcare institutions is limited [34].

### 6.4. Nanotechnology and Next-Generation Sequencing (NGS)

Nanotechnology has enormous potential for HIV diagnostics, with nanoscale sensors enabling the ultra-sensitive detection of virus particles. When combined with NGS, nanotechnology-based technologies may identify trace levels of HIV nucleic acids, even during acute infection phases, and track viral changes that confer medication resistance. These technologies provide comprehensive HIV genotyping, which allow accurate treatment regimens and real-time monitoring, making the treatment a success [52].

### 6.5. Integrative Platforms and Telemedicine

With the growth of digital health, integrated diagnostic platforms paired with telemedicine can let patients receive continuous HIV care remotely. These systems intend to deliver diagnostic findings directly to patients or healthcare practitioners, followed by teleconsultations for treatment planning. This method is especially useful for persons living in remote regions or who are hesitant to attend clinics in person owing to social stigma. As telemedicine becomes more incorporated into HIV care, it has the potential to close gaps in follow-up and adherence, resulting in better patient outcomes [53].

### 6.6. Future Directions: Emphasis on Management

The future of HIV diagnostics should focus on enhancing clinical management by monitoring disease progression, detecting drug resistance, and optimizing antiretroviral therapy (ART) [33]. Advanced tools like ultrasensitive assays and next-generation sequencing support long-term care, treatment adherence, and public health surveillance, ensuring a broader impact on patient outcomes and global HIV control efforts [54].

### 6.7. Future Directions

Future advances will most likely focus on extending diagnostic availability, improving mobility, lowering prices, and incorporating HIV tests into general healthcare processes. Continued multidisciplinary research, supporting public health policies, and infrastructure expenditures will be critical to ensure that new technologies reach those in need and advance the worldwide HIV pandemic control effort.

## 7. Conclusions

Innovative diagnostic techniques, such as CRISPR-based tools, AI applications, and lab-on-a-chip technologies, are changing HIV care by bridging the gap between fundamental research and clinical practice. These technologies offer more accuracy, faster outcomes, and broader accessibility, particularly in resource-constrained environments. However, hurdles persist, including logistic, economic, and educational constraints that must be overcome in order to completely incorporate these technologies into global HIV treatment systems. Strategic collaborations and investment are required to guarantee that these innovations reach those who need them the most, thereby contributing to the broader objective of ending the HIV pandemic and improving patient outcomes.

## Figures and Tables

**Table 2:** This table illustrates a comprehensive approach to overcoming logistic barriers that often complicate HIV diagnostic services [5,36,37].

Managing Logistical Barriers in HIV Diagnostics						
Identify Barriers	Resource Allocation and Prioritization	Supply Chain Optimization	Training and Capacity Building	Quality Assurance and Monitoring	Data Management and Reporting	Evaluate and Adapt
**Input**	**Input**	**Input**	**Input**	**Input**	**Input**	**Input**
List of logistical challenges (e.g., limited transportation, distribution issues, lack of trained personnel, equipment shortages).	Identified barriers and available resources (funding, personnel, and equipment).	Data on local supply chain challenges (supplier delays, regulatory requirements).	List of required skills and training gaps among healthcare personnel.	Established protocols for quality control and diagnostic standards.	Patient and diagnostic data, resource usage, and operational reports.	Ongoing data from monitoring, quality checks, and resource use.
**Process**	**Process**	**Process**	**Process**	**Process**	**Process**	**Process**
Conduct needs assessment in target regions, examining barriers specific to transportation, infrastructure, and resources.	Prioritize high-need regions or facilities, allocating resources based on severity of constraints and population needs.	Partner with local suppliers, optimize routes, and work with logistics experts to streamline transport and distribution.	Develop tailored training programs for operating diagnostic equipment, sample handling, and patient data management.	Implement routine quality checks and real-time monitoring using digital tools to track diagnostic accuracy and service delivery.	Use centralized databases to track results, manage patient information, and analyze logistical performance.	Review and assess logistical strategy effectiveness, adjust resource allocation, training, or supply chain processes as needed.
Output	Output	Output	Output	Output	Output	Output
Detailed report of logistical constraints by region or facility.	Resource allocation plan for phased implementation.	Optimized distribution routes and schedules.	Trained workforce capable of handling diagnostic procedures and equipment efficiently.	Quality-controlled processes and reliable diagnostics, with performance data for continuous improvement.	Comprehensive reporting on diagnostic impact, resource use, and areas for logistical refinement.	Updated logistics model with continuous improvements for scaling and replicating in other regions.
